# EPHX2 Inhibits Colon Cancer Progression by Promoting Fatty Acid Degradation

**DOI:** 10.3389/fonc.2022.870721

**Published:** 2022-03-30

**Authors:** Yiran Zhou, Xiao Li, Aoran Guan, Haodong Zhou, Yankun Zhu, Ruotian Wang, Ruhong Li

**Affiliations:** ^1^ Department of General Surgery, Yan’an Hospital Affiliated to Kunming Medical University, Yan’an Hospital of Kunming City, Kunming, China; ^2^ Key Laboratory of Tumor Immunological Prevention and Treatment of Yunnan Province, Kunming, China

**Keywords:** EPHX2, CRC, metabolic reprogramming, fatty acid degradation, ROS, lipid droplet

## Abstract

Tumor cells use metabolic reprogramming to keep up with the need for bioenergy, biosynthesis, and oxidation balance needed for rapid tumor division. This phenomenon is considered a marker of tumors, including colon cancer (CRC). As an important pathway of cellular energy metabolism, fatty acid metabolism plays an important role in cellular energy supply and oxidation balance, but presently, our understanding of the exact role of fatty acid metabolism in CRC is limited. Currently, no lipid metabolism therapy is available for the treatment of CRC. The establishment of a lipidmetabolism model regulated by oncogenes/tumor suppressor genes and associated with the clinical characteristics of CRC is necessary to further understand the mechanism of fatty acid metabolism in CRC. In this study, through multi-data combined with bioinformatic analysis and basic experiments, we introduced a tumor suppressor gene, EPHX2, which is rarely reported in CRC, and confirmed that its inhibitory effect on CRC is related to fatty acid degradation.

## Introduction

Colorectal cancer (CRC) accounts for approximately 10% of all annual cancer diagnoses and cancer-related deaths worldwide (1). It is the second most common cancer diagnosed in women and the third most common in men. Dysfunction of cellular energy metabolism is an important feature of almost all cancers. The metabolic differences between tumor and nontumor cells may be targeted for treatment (2). Targeting tumor metabolism remains an attractive strategy for anticancer therapy. However, the development of therapies targeting cancer cell metabolism has lagged significantly behind those targeting cell signaling pathways and immunotherapy (3). This situation is largely due to the difficulty in identifying highly specific enzyme inhibitors of bioenergy pathways. Cancer metabolism is highly dynamic and heterogeneous, and current analytical platforms limit our understanding of this complexity (4–6). Recently, metabolic reprogramming has been recognized as an important factor in tumor metabolic complexity (7). Metabolic reprogramming in cancer is largely regulated transcriptionally by oncogenes and mutated tumor suppressor genes (8). In addition to the classic aerobic glycolysis of tumor cells (Warburg effect), the regulation of lipid metabolism in tumor progression has been extensively studied (9). However, most current studies have focused on adipogenesis; the association between fatty acid degradation and cancer cells has received little attention (10, 11).

Fatty acids are mainly degraded by fatty acid oxidation (FAO). In addition to mitochondria, some fatty acids in animals are oxidized by peroxisome, such as occurs in β-oxidation of very long chain fatty acids (VLCFA) and α-oxidation and β-oxidation of long-branched chain fatty acids (12). Peroxisome is an important organelle involved in cell reduction–oxidation balance. The imbalance of lipid metabolism directly leads to cellular oxidative stress (13, 14). EPHX2 consists of two domains separated by a short linker rich in proline, encoding epoxide hydrolase (soluble epoxide hydrolase, SEH). SEH is a bifunctional enzyme whose C-terminal domain exhibits epoxide hydrolase activity that converts epoxide to corresponding diols, whereas the N-terminal domain exhibits phosphatase activity of lipid phosphate hydrolysis (15, 16). Only a few studies have reported on the biological role of EPHX2 in tumors, by its participation in lipid metabolism. Using bioinformatics, this study found that EPHX2 is an important gene that inhibits the progression of CRC, predicted its association with lipid metabolism, and verified the rationality of this metabolic model through experiments.

## Materials and Methods

### Data Mining

Data were obtained from COAD level 3 transcriptome sequencing data in the TCGA database. The protein assembly data and protein relative abundance details were obtained from the CPTAC database. The GMT file construction of specific pathway-related genes was obtained from the KEGG database. Bioinformatic analysis of the obtained data was performed using the R package (Limma, WGCNAC, ConsensusClusterPlus, and GSVA GSEAbase), GSEA 4.1 (http://www.gsea-msigdb.org/gsea/index.jsp), String online tool (https://cn.string-db.org/), and Cytoscape (https://cytoscape.org/index.html).

### CRC Tissue Microarray and Immunohistochemistry

Human CRC tissue microarrays consisting of 79 pairs of tumors and adjacent normal tissues were obtained from Shanghai Outdo Biotech, China. The anti-EPHX2 antibody was purchased from ProteinTech (Ag1283). Intensity score was in accordance with the following criteria: 0, no appreciable staining; 1, weak staining; 2, moderate staining; and 3, strong staining. The percentage score was based on the percentage of CRC positive cells (0–100). The immunohistochemical (IHC) staining was scored by two independent pathologists, and the final IHC score was calculated by multiplying the staining intensity score and positive staining percentage score. IHC scores ranged from 0 to 12 and were graded (low, 0–4 points; medium, 5–8 points; and high, 9–12 points) as described previously (12). Paired t-test was used to compare H score between tumor tissues and adjacent normal tissues.

### Cell Culture

The normal human intestinal epithelial cell line (NCM460) and human colonic carcinoma cell lines (HCT116, HT29, HCT15, and LOVO) were purchased from the Culture Collection of Chinese Academy of Sciences (Shanghai, China). In short, the HCT116 and HT29 cell lines were maintained in McCoy’s 5A medium (iCell, China). NCM460 was maintained in DMEM (Gibco, USA), and HCT15 was maintained in RPMI 1640 medium (Gibco). LOVO was maintained in the F12k medium (iCell, China). All media were supplemented with 10% fetal bovine serum (Gibco), supplemented with 1% penicillin and streptomycin (Gibco). All cell lines were cultured in a humidified incubator containing 5% CO_2_ at 37°C.

### RNA Extraction and Quantitative Real-Time Polymerase Reaction

Total RNA was isolated using the TRIzol reagent (Invitrogen, USA), following the manufacturer’s protocol, and RNA purity was detected using a NanoDrop 2000 spectrometer (Thermo Fisher Scientific, USA). The quantitative real-time polymerase chain reaction (PCR) was performed using SuperReal PreMix Plus (Invitrogen) in a StepOnePlus Real-time PCR Detection System (Applied Biosystems, CA, USA). Relative gene expression was calculated using the 2^-△△Ct^ method. Human GAPDH was used as an endogenous control for mRNA expression in the analysis.

### Vector Construction, Stable Transfection, and siRNA Interference Assays

Two groups, the EPHX2-overexpressing lentivirus vector (EPHX2-OE group) group and negative control vector (EPHX2-NC group) group, were established for the experiments. Tissues with EPHX2 overexpression were co-transfected with siRNA-EPHX2 (EPHX2-OE-siRNA group) or siRNA-negative control (EPHX2-OE-siRNA_NC group). The EPHX2-overexpressing lentivirus vectors were designed and constructed by the OBiO Company (Shanghai, China). CRC cells (HTC116), infected with the lentivirus, were screened continuously for 2 weeks using 2 µg/mL puromycin. After the efficacy verification of EPHX2 overexpression, the lentivirus-infected colonic carcinoma cells were used in subsequent steps of the experiments.

### Western Blotting

The proteins were extracted from cells, boiled with RIPA buffer (Beyotime, Shanghai, China), loaded and separated on a 10% SDS-PAGE gel (EpiZyme, Shanghai, China), transferred onto polyvinylidene fluoride membranes, and then incubated with the following primary antibodies: anti-EPHX2 (ProteinTech, Catalog number:10833-1-AP, USA) and anti-β-actin (abclonal, Catalog number: AC026, China). Protein expression was detected using the ECL reagent. β-actin was used as control. Three biological replicates were performed in each experiment.

### Annexin/Propidium Iodide Assay

Annexin V–IF647/propidium iodide (PI) apoptosis assay was performed using an Annexin V–IF647/PI kit (Servicebio, China). The cells in each group were subsequently incubated with 5 μL of Annexin V–IF647 and 5 μL of PI solutions at 37°C for 10 min in the dark. The number of apoptotic cells was measured using a flow cytometer (BD Biosciences Co. Ltd., USA). Three biological replicates were performed in each experiment.

### Transwell Invasion Assay

After lentiviral transfection, colonic carcinoma cells were seeded onto the basement membrane matrix (EC matrix, Chemicon, CA, USA) present in the insert of a 24-well culture plate. Thereafter, a complete culture medium was added to the lower chamber as a chemoattractant. After 48 h, the non-invading cells and EC matrix were gently removed with a cotton swab. The invasive cells located on the lower side of the chamber were fixed with 4% paraformaldehyde, stained with crystal violet, air-dried, and photographed with a microscope (Mshot, China). Three biological replicates were performed in each experiment.

### RNA Sequencing

Total RNA of HC116-GL120 and HCT116-H21064 were extracted using the TRIzol reagent (Invitrogen, CA, USA), following the manufacturer’s instructions. RNA sequencing was performed on an Illumina NovaSeq 6000 system of LC Sciences (USA). FastQC software (https://github.com/OpenGene/fastqc) were used to remove the reads that contained adaptor contamination, low quality bases and undetermined bases with default parameter. Then sequence quality was also verified using FastQC. We used HISAT2 (https://ccb.jhu.edu/software/hisat2) to map reads to the reference genome of *Homo sapiens* GRCh38.The mapped reads of each sample were assembled using StringTie (https://ccb.jhu.edu/software/stringtie) with default parameters. Then, all transcriptomes from all samples were merged to reconstruct a comprehensive transcriptome using gffcompare (https://github.com/gpertea/gffcompare/). After the final transcriptome was generated, StringTie and was used to estimate the expression levels of all transcripts. StringTie was used to perform expression level for mRNAs by calculating FPKM (FPKM = [total_exon_fragments/mapped_reads(millions) × exon_length(kB)]). The KEGG signal pathway enrichment analysis of FPKM data obtained by using GSEA4.1 software.

### Oil Red O Staining

The cell coverslips were first washed with phosphate-buffered saline (PBS), subsequently fixed with 4% paraformaldehyde for 20 min, washed three times with PBS, and incubated with 60% isopropyl alcohol for 10 s, followed by staining with freshly prepared 60% Oil Red O solution (100% solution: 0.5 g of Oil Red O dissolved in 100 mL of isopropylene) for 30 min and washing with PBS. The samples were counterstained with hematoxylin. Three biological replicates were performed in each experiment. The cell climbing pieces were scanned using a Motic Easy Scan (Motic, China) and analyzed using the image-Pro plus 6.0 software (https://www.xrayscan.com/software-image-pro-plus/).

### Measurement of ROS Levels

A cellular ROS assay kit (cat. no. ab186027; Abcam) was used to determine ROS levels. In a short, the transfected cells were plated (4 × 10^4^/100 µL per well) in a 96-well plate overnight. Subsequently, the cells were stained with ROS red stock solution for 30 min at room temperature. The absorbance was measured using a microplate reader, and ROS levels were determined by fluorescence increase at Ex/Em = 520/605 nm. Three biological replicates were performed in each experiment.

## Results

### Tumor Suppressor Gene Module of CRC

**Figure 1:**
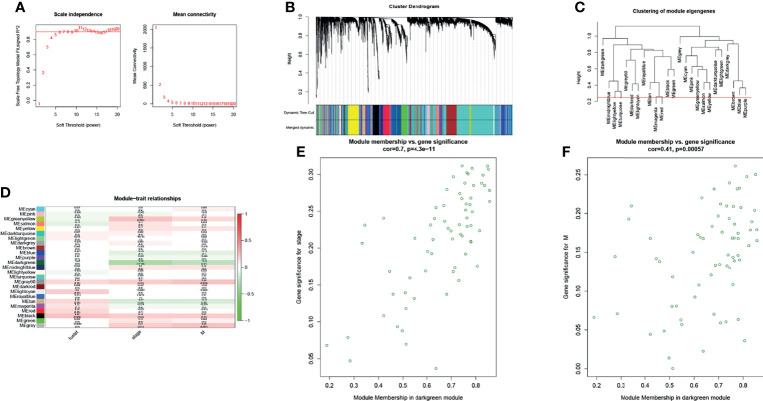
The WGCNA R package was used for finding clusters (modules) of highly correlated genes. **(A)** Scale-free fitting index and the average connectivity at different soft threshold powers were analyzed. In summary, 5 was the most suitable power. **(B)** The co-expression module was created. Each branch represents a gene, and each color below represents a co-expression module. **(C)** Cluster dendrogram of module eigengenes. **(D)** Heatmap analysis of the correlation between module characteristic genes and traits showed that the dark green module negatively correlated with stage and distant metastasis of patients with CRC (absolute value of cor >0.2, *p <* 0.05). **(E, F)** Scatter plot of dark green module members (MM) and gene significance (GS). A significant correlation was found between GS and MM (cor >0.4, *p < *0.0001).

Weighted correlation network analysis (WGCNA) was used to analyze RNA sequencing data (level 3) from 388 tumor samples from TCGA-COAD to assess the correlation between gene modules and patient survival, tumor TNM stage, and distant metastasis (**Figure 1**). The dark green module was mostly associated with the clinical characteristics of CRC through WGCNA, and the genes of this module may have a role in inhibiting the progression of CRC.

### Target Gene Set

**Figure 2:**
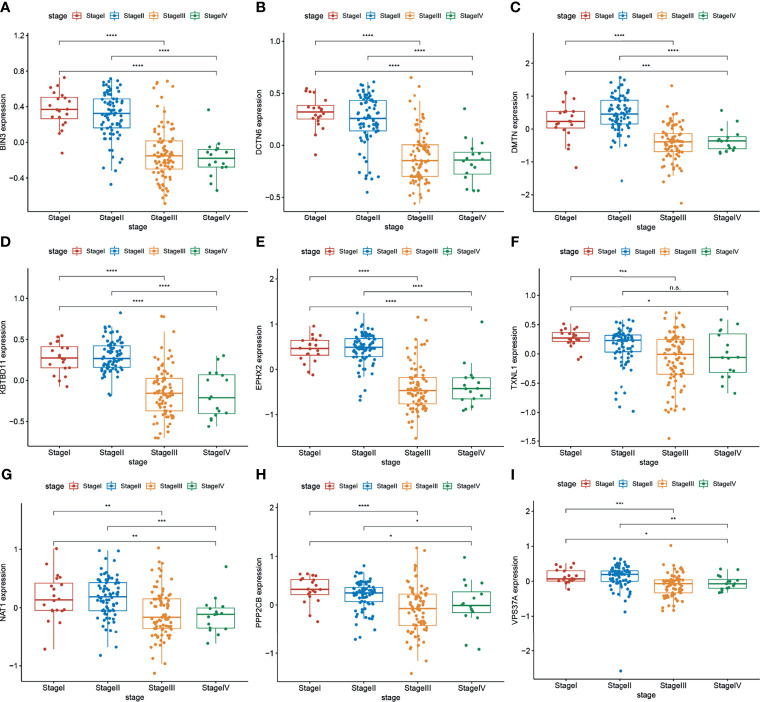
**(A–I)** Protein expression levels of target genes (BIN3, DCTN6, DMTN, KBTBD11, EPHX2, TXNL1, ANAT1, PPP2CB, and VPS37A) were negatively correlated with the TNM stage of CRC (n = 297 (stage I: 28, stage II: 120, stage III: 125, stage IV: 24). Wilcoxon’s test was used to compare groups. n.s, not significant; *p < 0.05,**p < 0.01,***p < 0.001,****p < 0.0001.

In bioinformatics prediction, the combination of mRNA and protein expression analyses of filtered data is beneficial to obtain more robust results. Therefore, the Colon Cancer Confirmatory Study (early release) protein assembly and protein relative abundance data (downloaded from the CPTAC database) were used to further analyze the genes in the dark green module. Genes with protein expression levels that were negatively correlated with TNM stage were used as the target gene set (**Figure 2**). A gene set of nine genes, which may inhibit the progression of CRC, were obtained.

### Clustering and Survival Analysis Based on the Target Gene Set

**Figure 3:**
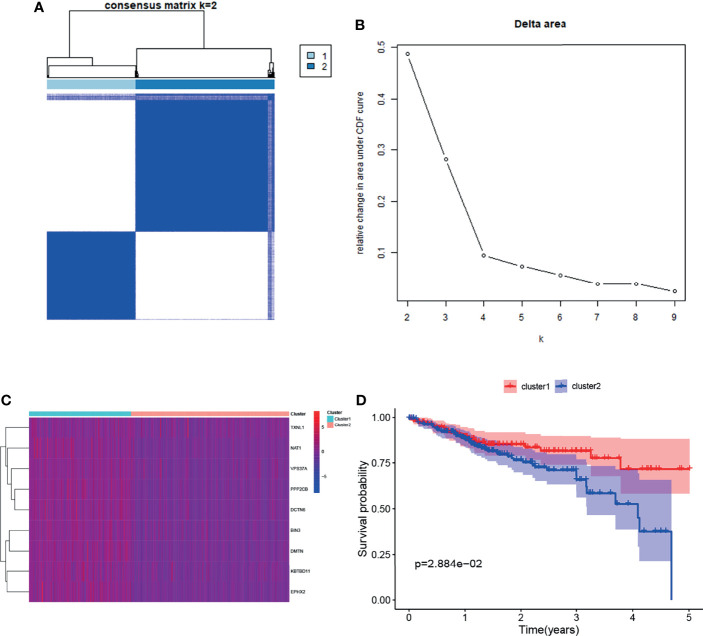
Clustering and survival analysis based on mRNA expression levels of target genes in the TCGA database. **(A, B)** Results of consistent cluster matrix, cumulative distribution function curve, and relative change in area under the curve showed that the expression of target genes could be divided into two types of CRC samples. **(C)** Heatmaps of gene expression in the cluster1 and cluster2 groups. **(D)** K–M survival curve showed that patients with cluster1 showed higher survival ability than those with cluster2 (*p < *0.05).

Cluster and survival analyses were used to further verify the role of target gene sets in CRC. Based on the mRNA expression levels of target genes set, the cluster analysis of 388 TCGA CRC samples was performed using the R package ConsensusClusterPlus. Consensus Clustering took a subsample from a set of microarray data and determined a cluster with a specified number of clusters (k). For each k, the paired consensus values (i.e., the proportion of the number of occurrences of two samples in the same subsample in the same cluster), were calculated and stored in a symmetric consensus matrix. Consensus matrices were summarized in several graphical presentations to enable the users to determine a reasonable number and membership of clusters. Cluster analysis was performed on 388 patients in the TCGA database according to the expression levels of target genes (Consensus Cumulative Distribution Function, CDF). The results showed that when k = 2, the consistency and clustering confidence was maximal. Furthermore, the survival analysis of patients with different cluster samples was performed. The Kaplan–Meier method was used to estimate the survival time distribution of different clusters, which was presented in the form of a survival curve to analyze the survival characteristics. The survival curve between both groups was tested using the log-rank test. The results showed that the survival rate of the cluster1 group was higher than that of the cluster2 group (*p <*0.05) (**Figure 3**). This further illustrates the correlation between the target gene set and inhibition of CRC progression.

### EPHX2 Was Regarded as the Core Gene of the Target Gene Set to Inhibit the Progression of CRC

**Figure 4:**
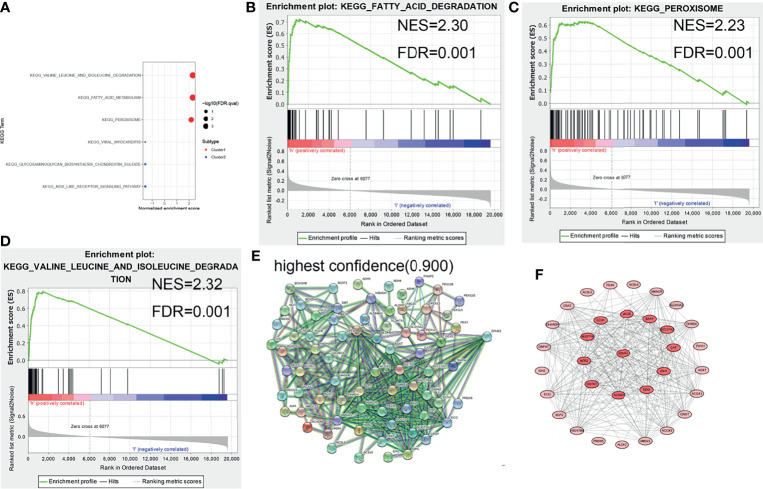
GSEA enrichment analysis and protein interaction network of target genes. **(A–D)** Three optimal enrichment signaling pathways were screened according to NES >2 and FDR <0.001. **(E)** PPI analysis of enrichment pathway genes and target genes was performed using the String online tool (highest confidence: 0.9). **(F)** MCC was used to screen for core node, and EPHX2 was the first target gene to appear in the core node (top 12).

The core genes in the target gene set were predicted. First, GSEA enrichment analysis was performed on tumor samples based on cluster grouping. According to the normalized enrichment score (NES) and the false discovery rate (FDR), three optimal enrichment KEGG pathways were obtained (NES >2 and FDR <0.001). Thereafter, protein–protein interaction (PPI) analysis of target genes and genes of the optimal enrichment pathway was performed using the String online database and Minimum Required Interaction Score set to the highest confidence (0.9), and disconnected nodes were hidden in the network. The maximal clique centrality (MCC) method was used to calculate the key proteins in the PPI network (using CytoHubba plug-in of Cytoscape software). Of the nine target genes, EPHX2 was the first key protein to appear (top 12) (**Figure 4**). First, the likely KEGG pathways of the target gene set were analyzed using GSEA. Subsequently, by PPI analysis, the first gene in the target gene set was selected and calculated as the core node. EPHX2 was considered to be the core gene involved in the inhibition of CRC progression.

### The Expression of EPHX2 Was Downregulated in CRC Tissues

**Figure 5:**
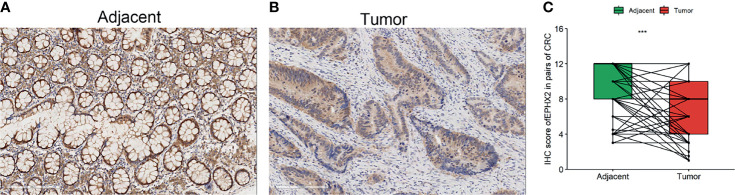
The protein expression profile of EPHX2 in human CRC tumor tissues (tumor) and adjacent normal tissues (adjacent). **(A)** The expression of EPHX2 in human CRC tumor tissues. **(B)** The expression of EPHX2 in adjacent normal tissues. **(C)** The expression of EPHX2 protein profile was significantly lower in tumor cells than in adjacent cells (paired t-test, n = 158, ***p < 0.001; original magnification, 200×; scale bars, 200 μm).

To investigate the expression of EPHX2 in patients with CRC, the protein expression profiles of EPHX2 in human CRC specimens and adjacent normal tissues were examined. Consistent with previous bioinformatic analysis, IHC analysis showed that EPHX2 expression was significantly downregulated in human CRC specimens, compared with adjacent normal tissues (**Figure 5**).

### EPHX2 Inhibits Invasion and Promotes Apoptosis of CRC Cells

**Figure 6:**
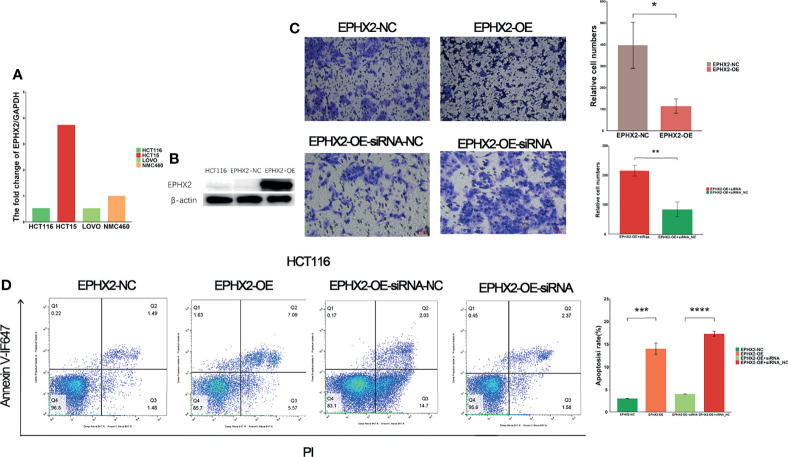
EPHX2 low expression cell line screening, cell transfection, and phenotypic determination. **(A)** HCT116 cells with low background expression of EPHX2 were selected for stable overexpression of EPHX2. **(B)** Detection of WB after stable transfection of EPHX2 overexpression into HCT116. **(C)** Invasion in the EPHX2 overexpression HCT116 cells was inhibited, and this effect can be reversed by EPHX2-siRNA co-transfection. ANOVA was used to compare the mean values of relative cell numbers between the two groups. *p < 0.05, **p < 0.01; original magnification, 10×; scale bars, 50 μm. **(D)** Cell apoptosis was promoted in EPHX2 overexpression group, and co-transfection with siRNA can reverse this effect. Statistical significance was determined with ANOVA. ***p < 0.001, ****p < 0.0001.

To verify the effect of EPHX2 on the progression of CRC at the cellular level, a CRC cell line (HCT116) stably transfected with EPHX2 overexpression (GL120) was constructed, and the transfection empty vector (H21064) was used as the negative control group (NC). The results showed that, compared with the NC group, overexpression of EPHX2 could inhibit the invasion of CRC cells and promote apoptosis. Moreover, EPHX2siRNA co-transfection could partially reverse the phenotype of cells (**Figure 6**).

### Cell Sequencing Analysis of the Molecular Mechanism of EPHX2 as the Core Gene of the Target Gene Set in Inhibiting the Progression of CRC

**Figure 7:**
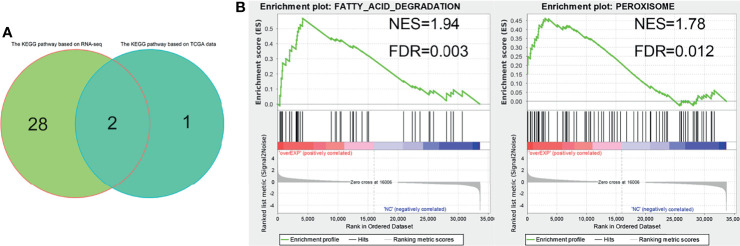
RNA-seq analysis of HCT116 cells stably transfected with EPHX2 overexpression. **(A)** Venn diagram ofoptimal enrichment KEGG pathway. **(B)** Finally, fatty acid degradation and peroxisome were labeled as EPHX2 as the core genes of the target gene set to inhibit the KEGG signaling pathway in the progression of CRC. The figure shows the results of cell RNA sequencing GSEA enrichment analysis after visualization using the ggplot2 R package.

We further analyzed the molecular mechanism of EPHX2’s inhibition of the progression of CRC at the cellular level and performed RNA-seq analysis on HCT116 cells stably transfected with EPHX2, with the NC group as control and three cell lines in each group. GSEA enrichment analysis was performed for both groups. The results were screened using FDR <0.05, and 30 candidate KEGG pathways were obtained. The optimal enrichment signal pathway of the previously obtained target gene set in the clinical samples of CRC was used for intersection analysis (**Figure 7A**). Finally, peroxisome and fatty acid degradation signal pathways, which may be the most likely KEGG pathways for EPHX2 to act as the core gene in the target gene sets in the inhibition of the progression of CRC, were labelled (**Figure 7B**).

### Evaluation of Immune Cell Content and Hypoxia Signaling Pathway Based on Cluster1 and Cluster2 Grouping

**Figure 8:**
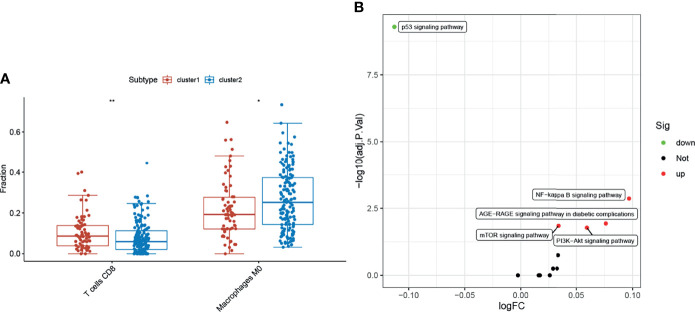
Evaluation of immune cell and hypoxia signaling pathways. **(A)** Compared with the cluster2 group, the cluster1 group had upregulated CD8 T lymphocytes, whereas M0 macrophages were downregulated (*p < 0.05, **p < 0.01). **(B)** Changes in cluster1 and cluster2 hypoxia signaling pathways were not significant (FDR <0.05 was considered statistically significant, but the maximum absolute value of logFC was <0.2).

Hypoxia is an internal environment in almost all solid tumors. Hypoxia interacts with fatty acid metabolism and can regulate tumor immunity (17, 18). Therefore, the differences in the immune cell content and hypoxia signal pathway between the cluster1 and cluster2 groups were evaluated. The CIBERSORT R package was used to analyze TCGA-COAD RNA-seq data and perform differential analysis for the cluster1 and cluster2 groups. The KEGG pathway database was used to obtain genes related to the hypoxia signaling pathway and create GMT files (**Supplementary Material 1**). The difference in hypoxia signal between the cluter1 and cluster2 groups was evaluated using the GSVA method. The expression of CD8T lymphocytes was upregulated in the cluster1 group, whereas the downregulation of M0 macrophage expression (**Figure 8A**) suggested that the target gene set may play a role in promoting the immunity of CRC. However, whether EPHX2 is directly involved in this biological effect remains to be further evaluated. No significant difference in hypoxia signal between cluster1 and cluster2 groups (**Figure 8B**) existed, which suggests that EPHX2 as a core gene inhibits colon cancer through peroxisome involvement in fatty acid degradation. Because it is not disturbed by the anoxic microenvironment of colon cancer, it cannot evaluate whether the hypoxic microenvironment of CRC is related to the expression of EPHX2.

### Overexpression of EPHX2 Increases the Content of ROS and Decreases the Formation of Lipid Droplets in HCT116 Cells

Enhancing fatty acid degradation to generate ATP helps cancer cells to resist glucose deprivation or hypoxia, but the production of large amounts of ROS can lead to chronic oxidative stress. Under long-term oxidative stress, cancer cells are more likely to initiate the cell death process than normal cells (19). Lipid droplets (LDS) are highly organized spherical organelles and a repository of cellular fatty acids (20). The anoxic environment of solid tumors can promote the formation of lipid droplets by inducing PLIN2, which helps cells to use them for antioxidant defense after reoxygenation (21). Compared with normal colon tissue, CRC cells have several lipid droplets (22, 23). In previous studies, minimal difference was present in hypoxia signal in CRC samples grouped according to the target gene set. Therefore, the mechanism of EPHX2’s inhibition of the progression of CRC was preliminarily verified by detecting ROS content and oil red staining in both groups.

**Figure 9:**
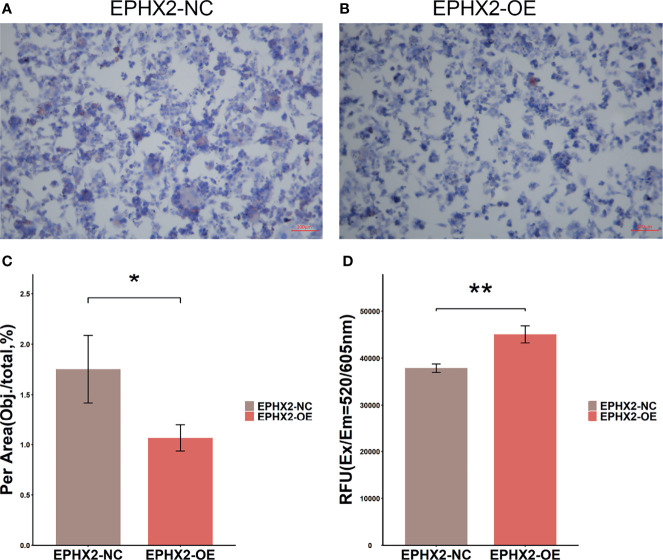
Lipid drop Oil Red O staining and detection of ROS in HCT116 cells. **(A)** Lipid drop Oil Red O staining in EPHX2-NC group. **(B)** Lipid drop Oil Red O staining in EPHX2-OE group. **(C)** The area (Obj./total: the red stain occupying the total area of visual field) was calculated using ImageJ pro plus6, which randomly selected high, medium, and low ORO staining images from each of the three repeated experiments. Statistical significance was determined with ANOVA. Compared with the EPHX2-NC group, the EPHX2-OE group had decreased ROS level. *p < 0.05; original magnification, 100×; scale bars, 100μm. **(D)** ROS in the two groups was detected using the enzyme labeling instrument. ANOVA was used to compare the mean values after absorbance conversion. Compared with the EPHX2-NC group, the EPHX2-OE group had increased ROS level, **p < 0.01.

In HCT116 cells, the content of lipid droplets in the OE group was lower than that in the NC group (*p <*0.01) (**Figures 9A–C**). Additionally, higher levels of ROS were present in cells (*p <*0.01) (**Figure 9D**). This observation preliminarily confirmed that EPHX2 could promote the degradation of fatty acids and increase the release of ROS in HCT116 cells.

## Discussion

Tumor cells show selective upregulation of transcription factors, which mediate complex internal signaling pathways and oncogenic pathways, and dysregulation of tumor suppressor genes according to environmental and genetic factors. These tumor cell activities lead to metabolic reprogramming, which is the key to maintaining the bioenergy production and reduction-oxidation homeostasis of the tumor (24, 25). Fatty acids play an important role in cellular metabolism by participating in the synthesis of biofilms and regulation of mobility. Additionally, they serve as second messengers in signaling pathways that maintain homeostasis and as a form of energy storage in animals (11, 26). The change in fatty acid metabolism is one of the most prominent metabolic changes in cancer. Increasing the synthesis or uptake of lipids contributes to the rapid growth of cancer cells and tumor formation (17). These studies have provided new insights for tumor fatty acid metabolism, but the complexity of fatty acid metabolism has not been fully explained. First, the clinical correlation between EPHX2 and CRC confirmed EPHX2’s role in inhibiting the invasion of CRC cells and promoting apoptosis. We found the clinical correlation between EPHX2 and CRC for the first time, confirmed its role in inhibiting the invasion and promoting apoptosis of CRC cells, and predicted that it may promote fatty acid degradation and inhibit the progression of CRC through peroxisomes. This may be an important supplement to the study of the relationship between the progression of CRC and metabolism.

EPHX2 encodes SEH, which is a key gatekeeper enzyme that affects the lipid signal function of a series of metabolites by decomposing epoxy fatty acids into corresponding diols (27, 28). SEH activity decreases the function of lipid signal transduction (29). The downregulation of EPHX2 expression is associated with poor prognosis and clinical characteristics of liver cancer and prostate cancer (30, 31). According to Panigrahy (32), EPHX2 inhibitors can promote the progression of melanoma and fibrosarcoma in mouse models by increasing the level of an endogenous lipid mediator, epoxy eicosatrienoic acid (EETs) (32), suggesting that a relationship exists between EPHX2-lipid metabolism and tumor progression. The N-terminal domain of SEH shows the activity of phospholipid hydrolysis. Phospholipids are an important component of the cell membrane, and the peroxidation of polyunsaturated fatty acids in phospholipids can lead to iron-dependent non-apoptotic cell death (iron ptosis) (33–35). Wang (36)’s determination of tissue liposomes in 20 patients with CRC showed that the liposomes in CRC were similar to those in normal colonic mucosa. The changes in lipid abundance were limited to several liposomes, such as lysophosphatidic acid and ether, that were involved in lipid signaling and ROS clearance (37). They were mainly degraded in peroxisomes, which are important organs of cellular oxidative stress. The liposome-mediated lipid catabolism affects the production of ROS, because the electron transport chain of mitochondria is the main location of their production. Cancer cells generally have higher levels of ROS to promote proliferation or invasion, but higher than the threshold level of ROS can strongly promote cancer cell apoptosis or iron death, thus inhibiting tumor progression (38–40). This dual effect of ROS on cancer cells must be considered when predicting the mechanism and characteristics of cancer cells’ response to lipid metabolism changes. The fatty acids obtained by endogenous synthesis or exogenous uptake of cancer cells are stored in lipid droplets in the form of triglycerides and cholesterol (23). Compared with normal colon tissues, CRC tissues have several lipid droplets, which protect cancer cells from the toxicity of excess ROS and free lipids. These lipid droplets are conducive for cell survival, invasion, and drug resistance (41, 42). The expression of lipid droplets is related to the high tumorigenicity of CRC stem cells (43). The results of oil red staining and total ROS content detection showed that in HCT116 cells overexpressing EPHX2, lipid droplets decreased, and total ROS content increased.

The above results preliminarily confirmed the mechanism of EPHX2’s inhibition of the progression of CRC by promoting fatty acid metabolism, but there are still many scientific problems in this metabolic process that need to be confirmed by further experiments, such as the scientific questions relating to the specific role of peroxisomes?function of mitochondria and ROS threshold for promoting apoptosis of CRC cells? Notably, fatty acid degradation produces large amounts of ATP, which is an important energy source for activating important tumor immune cells, such as CD8 T lymphocytes. Furthermore, fatty acid degradation is thought to enhance its immune memory effect (44–46). We detected the changes in immune cells in cluster1 group and cluster2 group using the CIBERSORT method (https://cibersort.stanford.edu). The results showed that the proportion of CD8T lymphocytes increased significantly in the cluster1 group (*p <*0 01), but the percentage of M0 macrophages decreased (*p <*0.05). However, few studies on the differentiation and mechanism of M0 macrophages in tumors were available, but patients with bladder cancer and hepatocellular carcinoma with low expression of M0 macrophages have relatively better clinical prognosis in gliomas. M0 macrophages have the malignant characteristics of tumor cells, which is related to the poor clinical prognosis of patients (47–49).

## Conclusion

The clinical correlation between EPHX2 and CRC was explored, the inhibitory effect of EPHX2 on the progression of CRC was confirmed, and a real lipid metabolism model of colon cancer with the participation of EPHX2 was preliminarily established. The model is an important supplement to the study of lipid metabolism in the progression of CRC.

## Data Availability Statement

The datasets presented in this study can be found in online repositories. The names of the repository/repositories and accession number(s) can be found below: https://www.ncbi.nlm.nih.gov/geo/query/acc.cgi?acc=GSE197535.

## Author Contributions

YRZ completed the bioinformatic analysis, statistical analysis of data, and draft manuscript. XL and HZ completed the cell transfection and cell phenotype identification experiments. AG and RW finished the oil red staining experiment and cellular ROS testing. YKZ was responsible for data collection. RL designed the study and modified the manuscript to form the final draft. All authors contributed to the article and approved the submitted version.

## Funding

This study was supported by special Fund for Basic Research of Yunnan Science and Technology Department (202101AU070122).

## Conflict of Interest

The authors declare that the research was conducted in the absence of any commercial or financial relationships that could be construed as a potential conflict of interest.

## Publisher’s Note

All claims expressed in this article are solely those of the authors and do not necessarily represent those of their affiliated organizations, or those of the publisher, the editors and the reviewers. Any product that may be evaluated in this article, or claim that may be made by its manufacturer, is not guaranteed or endorsed by the publisher.
